# Chronic Diseases and Mortality in Canadian Aboriginal Peoples: Learning From the Knowledge* *This article is part of a joint publication initiative between *Preventing Chronic Disease* and *Chronic Diseases in Canada*. *Preventing Chronic Disease* is the secondary publisher, while *Chronic Diseases in Canada* is the primary publisher.


**Published:** 2010-12-15

**Authors:** M. King

**Affiliations:** Canadian Institutes of Health Research, Institute of Aboriginal Peoples’ Health

It is a sad fact that Canada’s Aboriginal people, whether living in rural communities or in urban centers, have a significantly lower life expectancy than non-Aboriginal Canadians.^1^ The gap in health status of Canada’s Aboriginal peoples is a matter of ongoing concern;^2^ recognizing and understanding the social determinants of health is key to understanding the difference in health status and, in my view, key to achieving success in addressing and correcting this problem. However, it is important to realize that there are unique social determinants for Aboriginal peoples associated with their cultures, histories and colonization, and the current social, economic, political and geographic context.^3^

Bruce et al.^4^ examined obesity and the related comorbidities—dyslipidemia, hypertension and diabetes—in a Manitoba First Nations community. As in other Aboriginal populations (e.g. in neighbouring Saskatchewan^5^), they found the prevalence of obesity and these comorbidities is higher in women than in men, and that the comorbidities are common even in young adults. Undiagnosed hypertension is also common. In the same Manitoba First Nations community, Riediger et al.^6^ found a high cardiovascular risk due to low plasma apolipoprotein A1 levels, particularly among women.

Ng et al.^7^ studied arthritis in the national Aboriginal population, focusing on the differences between the northern territories and the 10 provinces in the south. They used data from the 2006 Aboriginal Peoples Survey (APS), a post-census survey conducted by Statistics Canada that includes health, social and economic questions. The APS covers Inuit and Métis, as well as First Nations living outside of Indian reserves; on-reserve First Nations are covered by the 2002/03 Regional Longitudinal Health Survey conducted by the Assembly of First Nations.^8^ Because of oversampling of northern residents in the APS, effective comparisons can be made with the more populated south. Self-reported arthritis and rheumatism is the most common medical condition reported by Aboriginal people in Canada. In the south, where more than 90% of the Aboriginal population live, the overall prevalence is 20.1% compared with 25.3% for on-reserve First Nations in the same geographic area.^8^ The prevalence of arthritis or rheumatism in the north is considerably less (12.7% overall), and is lower for both First Nations and Inuit in comparison with the south. A higher proportion of individuals with arthritis report at least one other chronic disease, and more people with arthritis consult health professionals, while fewer—perhaps not surprising—are employed, although cause and effect was not established.

Tjepkema et al.1 looked at mortality of urban Aboriginal adults over an 11-year period (1991–2001), linking mortality registry data with census data and data on tax filers to achieve an urban Aboriginal cohort of some 25 500 among a total urban cohort of 2.6 million. The main variable, remaining life expectancy at age 25 years, is significantly lower for Aboriginals living in urban centers than for urban non-Aboriginal Canadians. For men, the life expectancy is 4.7 years lower; for women, 6.5 years. These differences are about the same as the overall figures that include on-reserve and non-urban residents recently published by the same researchers;^9^ these also show the same gender bias, i.e. the gap in life expectancy is greater for Aboriginal women by about 2 years.^9^ The researchers found that the two leading causes of mortality in Aboriginal Canadians are circulatory system diseases and cancers, the former being elevated for both men and women compared with the non-Aboriginal population, the latter only for Aboriginal women.^1^ Many specific causes of death are remarkably elevated, particularly alcohol-related causes and external causes, including suicides and accidents; deaths amenable to medical intervention are elevated in both men and women.^1^

These descriptions and understandings of the health gap and risk factors for chronic diseases in Canada’s Aboriginal populations are important, but we need to understand all the causes and then move on to interventions that will address, prevent and reverse the problems. The four studies described here all take their fields of interest from the mere descriptive to understanding the physical, socioeconomic and societal risk factors. The next step, and perhaps the crucial one, is how to use the knowledge to correct the disparity. The study by Bruce et al.^4^ ultimately offers hope: the community is engaged in projects to address, prevent and perhaps even reverse the otherwise inevitable increasing obesity predicted by the risk factors. Few details are given, but we are told that a project aiming to prevent gestational diabetes through controlling weight gain during pregnancy is under way; that the community operates a fitness center; that the health center offers education on diet, exercise and wellness; and that walking groups have been organized, as well as activity programs in the schools. In all of this, the investigators continue to work with the community. It is unfair to expect the same degree of follow-up with national survey data because action to utilize health information and transform behaviours and conditions generally requires local community engagement. However, it is important to work with national organizations to transform the health knowledge into action and policy.

The Canadian Institutes of Health Research (CIHR) embraces the need to reduce health inequities in Aboriginal peoples as one of its main strategic directions.^10^ The CIHR Institute of Aboriginal Peoples’ Health (IAPH) supports community engagement, capacity building and network development as key platforms towards achieving health equity for Aboriginal peoples, and will continue to do so. But we need to move beyond this into the realms of intervention research (what worked, what didn’t, and why) and knowledge translation (transformation to different contexts and scaling up) to define the models of good practice that will enable our communities to achieve their goals of health equity. Since health equity can never be achieved without the wholeness that includes the mental, physical, emotional and spiritual parts of our lives, we must go beyond the conventional social determinants of health to look at factors that include promotion of resilience through spirituality, culture, language revitalization, traditional activities and other forms of cultural connectedness.^3^ With follow-up by researchers and with local, regional and national community engagement, we hope to eventually eliminate the gap in health status that divides Canada’s Aboriginal peoples from the non-Aboriginal mainstream.
